# A Case of Childhood Acute Lymphoblastic Leukemia With Retinitis Pigmentosa-like Fundus Findings

**DOI:** 10.7759/cureus.77094

**Published:** 2025-01-07

**Authors:** Nami Okujima, Hirohito Iimori, Atsushi Shiraishi

**Affiliations:** 1 Department of Ophthalmology, Ehime University School of Medicine, Toon, JPN

**Keywords:** acute lymphoblastic leukemia, car-t therapy, molecular remission, monitoring, retinal changes

## Abstract

Ophthalmic manifestations are a common occurrence in leukaemia cases and may result from a number of factors, including direct leukemic infiltration, haematologic abnormalities, central nervous system (CNS) involvement, or treatment-related effects. This case report discusses a paediatric patient with acute lymphoblastic leukaemia (ALL) who developed recurrent ocular symptoms, including retinitis pigmentosa-like findings, which is degeneration with pigment neopigmentation in the mid- to peripheral fundus, during remission, underscoring the necessity of continuous monitoring. A four-year-old female with a history of ALL, diagnosed at two years and three months of age, presented with ophthalmic complications during remission. The leukaemia was characterised by positivity for CD20, CD19, and the mature B-cell antigen, as well as a KMT2A-AFF1 gene rearrangement. Despite the administration of an intensive chemotherapy regimen and CAR-T cell therapy, the patient experienced a relapse and subsequently underwent umbilical cord blood transplantation, resulting in the attainment of molecular remission. However, at three years and seven months of age, a notable increase in the size of the left orbital tumour was observed. At three years and 11 months of age, the patient exhibited retinal changes that were consistent with retinitis pigmentosa, as confirmed by fundus photography. Additional findings included moderate left pupil dilation, an absent light reflex, optic disc swelling, dilated retinal vessels, and a serous retinal detachment. The presence of KMT2A-AFF1 mRNA in the anterior chamber fluid served to confirm the occurrence of an ocular relapse, a finding that was also evident in the bone marrow analysis. The patient was treated with salvage chemotherapy and localised radiation therapy (24 Gy) to the left eye, resulting in partial resolution of symptoms. At the four-year and three-month follow-up, retinitis pigmentosa-like changes with proliferative alterations persisted, although the serous retinal detachment had resolved. This case demonstrates that ophthalmic manifestations can occur even during the remission phase of leukaemia, emphasising the importance of regular, multidisciplinary follow-up.

## Introduction

Leukaemia is defined as a clonal proliferation of haematopoietic stem cells in the bone marrow [1]. Acute lymphoblastic leukaemia (ALL) is a common occurrence in paediatric patients [1]. The aetiology of leukaemia is multifactorial, encompassing both genetic predisposition and environmental factors such as exposure to ionising radiation [1]. The clinical manifestations of leukaemia are non-specific and may include fever, fatigue, weight loss, bone pain, bruising or bleeding [1]. Ocular manifestations are common in patients with leukaemia [2]. They may be caused by direct tumour cell invasion, haematologic abnormalities, central nervous system involvement, opportunistic infections, or indirect or secondary causes related to treatment [3]. They are clinically important because they may precede a systemic diagnosis or be a sign of relapse [3]. In addition, ocular manifestations associated with leukaemia are more common in acute and myeloid cases and less common in chronic and lymphoid subtypes [4]. Furthermore, the incidence of ocular manifestations in acute leukaemia has been reported to be higher in adults than in paediatric patients [5].

A systematic review of all existing data on clinical manifestations was conducted to estimate the frequency of signs and symptoms present at or before diagnosis, with the aim of assisting clinicians in early detection. The analysis included 3,084 individuals from 33 studies and concluded that, even in remission, leukaemia can present with a variety of symptoms, including fever, anaemia, petechiae, bone and joint pain [6]. Therefore, careful follow-up is essential.

This case report describes a case of childhood ALL in remission with recurrent ocular symptoms. The patient presented with retinitis pigmentosa-like fundus findings during the remission phase of ALL and was treated with a combination of chemotherapy and radiotherapy and is still under observation.

## Case presentation

This report details a case of paediatric ALL presenting with distinctive retinal pigmentary degeneration-like findings. The patient was a four-year-old girl who initially presented with ALL at the age of two years and three months. She was positive for CD20 and 19, positive for mature B-cell antigens, as well as the KMT2A-AFF1 gene, which is associated with unfavourable outcome and increased risk of extramedullary lesions. Despite the administration of standard remission induction therapy, the patient exhibited no response and was subsequently treated with blinatumomab.

**Figure 1:**
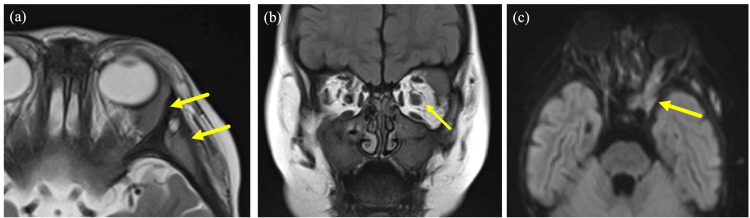
MRI image at two years and 10 months (a) T2-weighted low-signal soft-tissue shadow is seen from the outer lateral wall of the left orbit to the temporal fossa and submental area of the temporal cortex. (b) The left optic nerve is enlarged. (c) The lesion extends along the optic nerve to the cavernous sinus.

At two years and 10 months of age, the patient presented with enlargement of extramedullary lesions, including those in the left orbit (Figure 1). The lesion in the left orbit was most prominent at two years and 10 months of age. By the age of three years and two months, due to the progression of the disease, the patient underwent chimeric antigen receptor (CAR) T-cell therapy, resulting in the achievement of molecular remission. At the age of three years and seven months, there was no observable tendency for the lesion to enlarge, and the fundus was observed to be clear. Unfortunately, the patient experienced a molecular relapse at the age of three years and nine months, and subsequently underwent umbilical cord blood transplantation, which once again resulted in the achievement of molecular remission.

**Figure 2:**
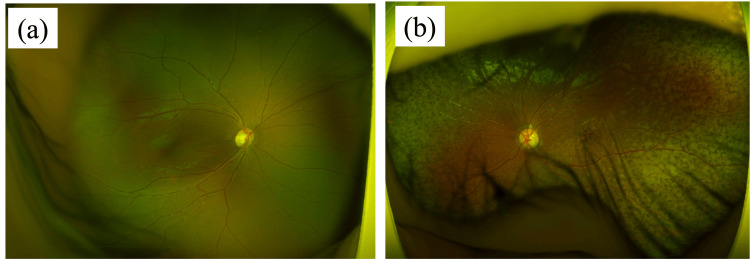
Fundus photographs at three years and 11 months (a) The right eye is depicted and no discernible abnormalities were evident. (b) The left eye is illustrated and retinitis pigmentosa-like findings were apparent.

**Figure 3:**
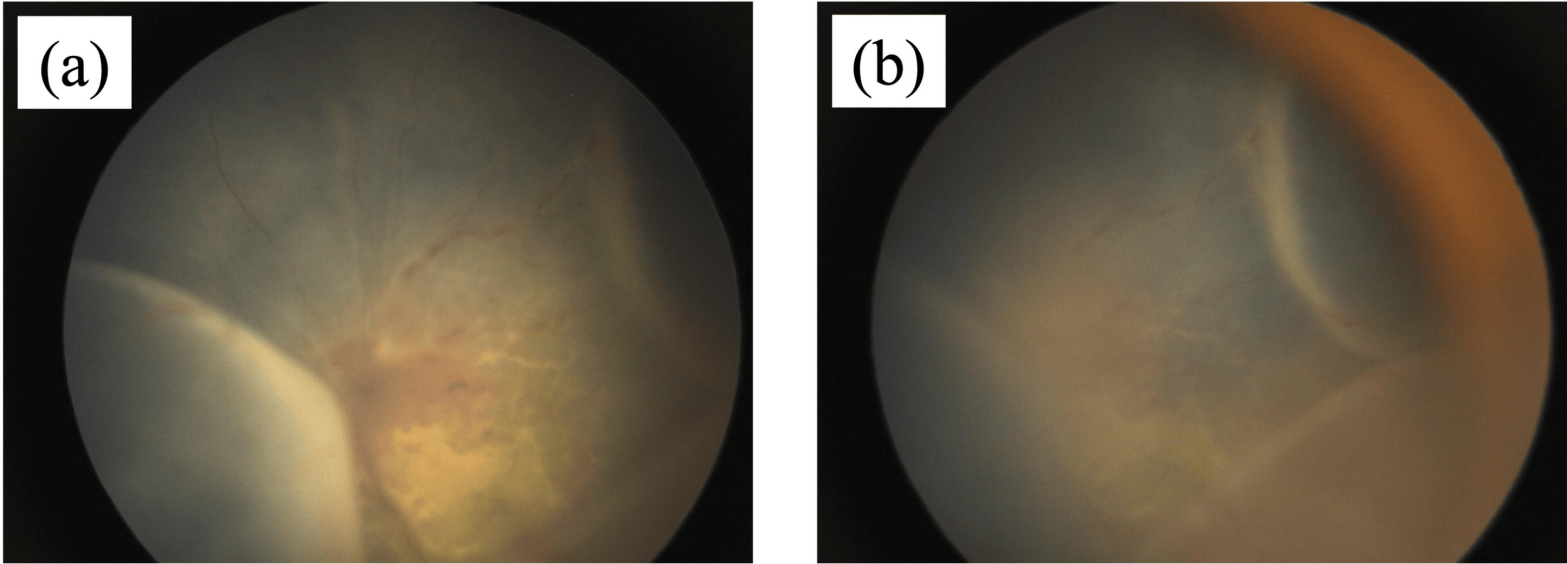
Fundus photographs at four years and two months Examination at four years and two months revealed moderately dilated left pupil and loss of light reflex. Fundus examination revealed optic nerve papillary swelling, dilated and tortuous retinal vessels, and serous retinal detachment.

During the course of her treatment, she exhibited notable ocular manifestations. At the time of diagnosis, no ocular abnormalities were observed. However, subsequent to this observation, symptoms indicative of ocular involvement manifested. At three years and 11 months, retinitis pigmentosa-like findings, which is degeneration with pigment neopigmentation in the mid- to peripheral fundus, were observed (Figure 2). These included moderate mydriasis with no light reflex in the left eye, optic disc swelling, tortuous and dilated retinal vessels, and serous retinal detachment, which were documented through fundus photography (Figure 3).

**Figure 4:**
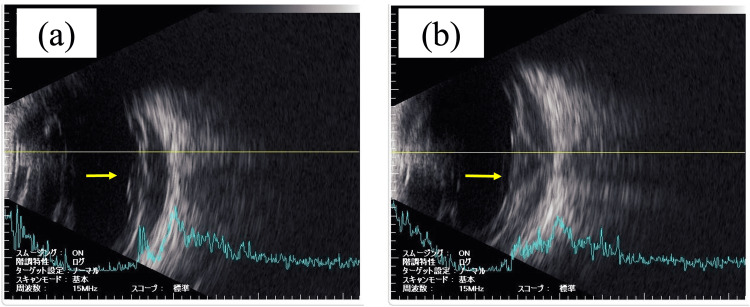
Ultrasonographic images at four years and two months (a) and (b) both show thick, hyperintense findings under the retina (not partially but entirely). (Two images are shown to illustrate that the hyperintense findings are not partial, but rather are seen throughout the entirety of the lesion.)

**Figure 5:**
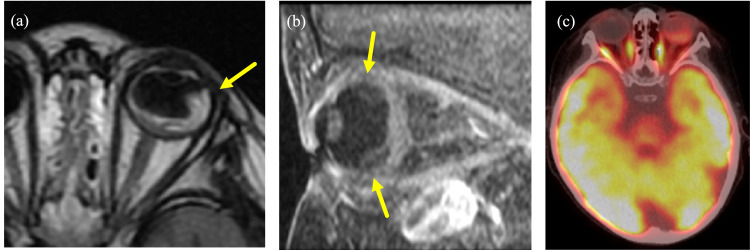
MRI and PET-CT images at four years and two months (a) and (b) demonstrate an elevated lesion and retinal detachment that are suspicious of leukaemic infiltration under the left retina. Additionally, (c) shows a banded fluorodeoxyglucose accumulation in the left retina, thereby strongly suggesting leukaemic retinal infiltration.

Subsequent examinations revealed a thick, hyperreflective subretinal lesion visible on ultrasound and confirmed by ultrasound and PET-CT scans (Figures 4, 5). These ocular manifestations prompted the initiation of targeted radiotherapy (24 Gy) to the left eye after KMT2A-AFF mRNA was detected in the anterior chamber fluid, confirming the recurrence of the left eye lesion.

**Figure 6:**
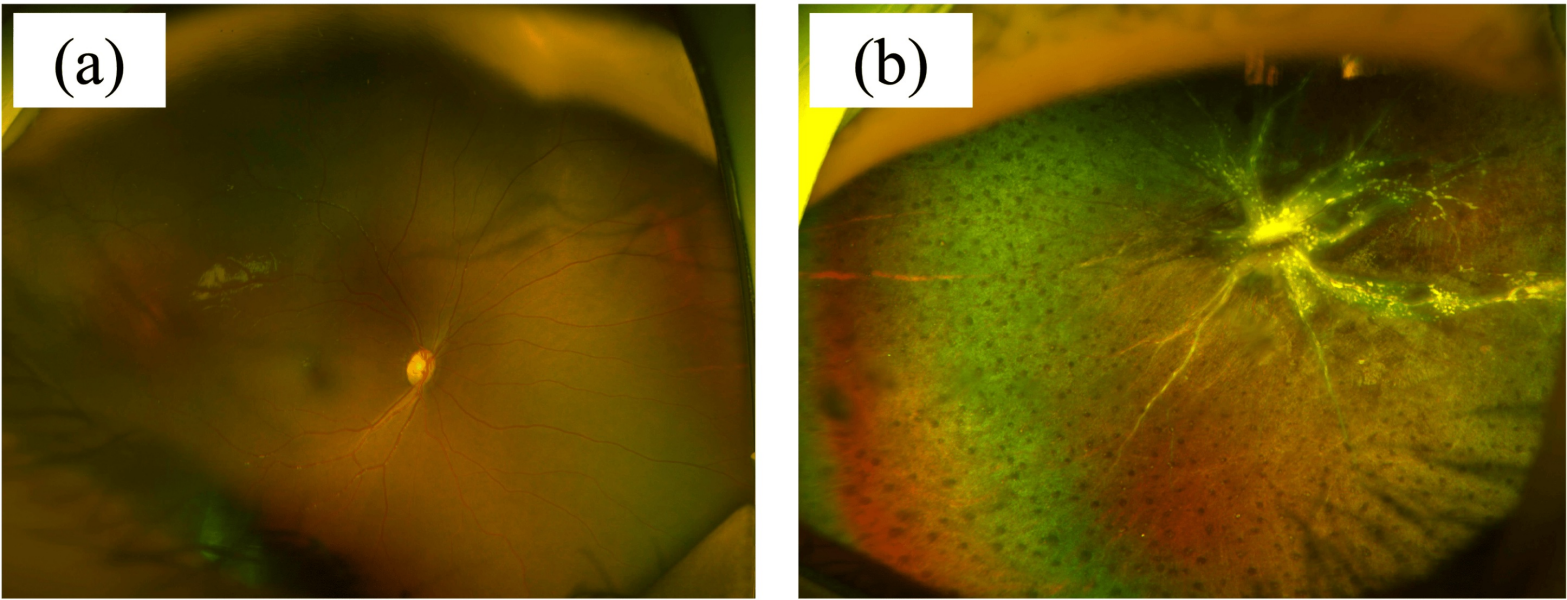
Fundus photograph after treatment (four years and three months) In the figure, (a) displays the image of the right eye, and no indications of retinitis pigmentosa were observed; (b) depicts the image of the left eye, wherein the retinal blood vessels exhibited cloudiness, and the serous retinal detachment dissipated. However, evidence suggestive of retinitis pigmentosa persisted, and proliferative alterations were evident.

**Figure 7:**
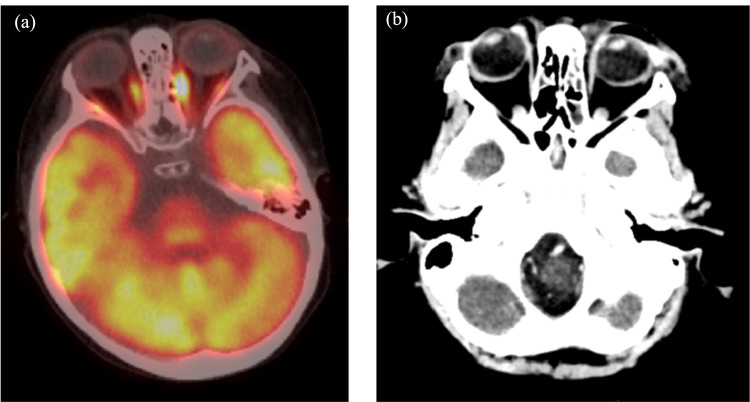
PET-CT and CT images after treatment (four years and three months) (a) PET-CT images showed that the fluorodeoxyglucose (FDG) accumulation in the left retina was obscured and considered to be a treatment effect. (b) CT images also showed no thickening lesions or detachment of the retina.

**Figure 8:**
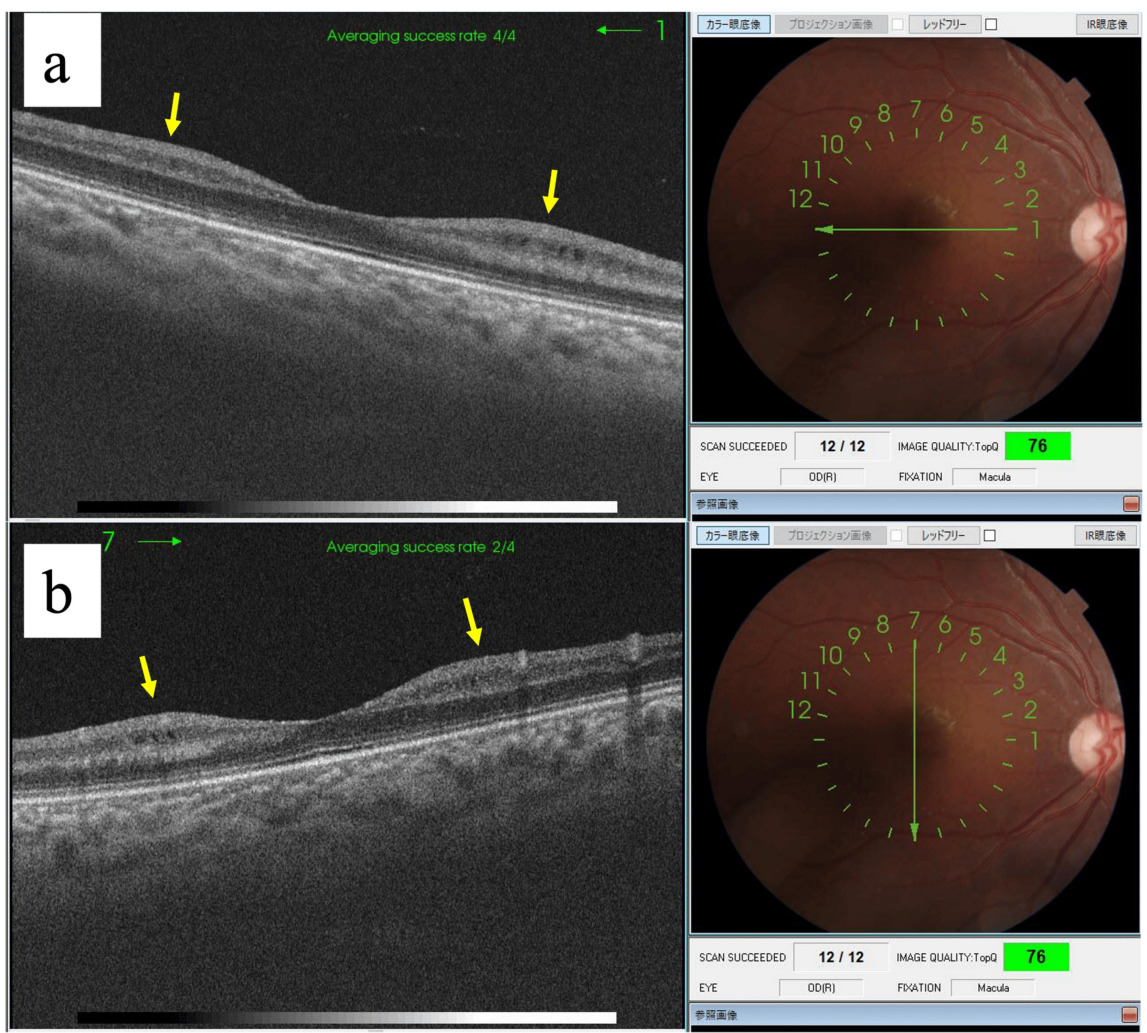
Optical coherence tomography (OCT) image at four years and eight months At four years and eight months, the right eye has no retinitis pigmentosa-like findings, but the optic nerve papilla is slightly pale and OCT shows intraretinal oedema. The cause of the inner layer oedema is unknown, but we are following her progress with caution. The patient has difficulty wearing glasses, and visual acuity tests have not been performed due to lack of cooperation.

Post-treatment observations at four years and three months showed resolution of the serous retinal detachment, although pigmentary and proliferative changes persisted, indicating residual disease activity (Figures 6, 7). The patient continued to receive systemic chemotherapy in addition to local ocular treatments. The patient continued to have ocular abnormalities, including vascular whitening and optic disc pallor in the right eye, along with retinal oedema, as documented by OCT at four years and eight months (Figure 8).

## Discussion

Two important lessons can be learned from this case. First, ocular manifestations, including retinitis pigmentosa-like findings, may occur during the remission of leukemia. Second, regular multidisciplinary follow-up is necessary to address various leukemia-related symptoms, including ocular manifestations.

In the present case, ocular manifestations occurred during the remission phase of ALL. However, previous studies have demonstrated that ocular manifestations frequently manifest in patients with leukaemia who are not in remission [7,8]. Also, this case presented with retinitis pigmentosa-like findings. Retinitis pigmentosa is a debilitating and progressive eye disease that affects more than 1 million people worldwide [9]. Previous studies have suggested that the causes of secondary retinitis pigmentosa are malignancy, drug-induced, and trauma [10-12]. Although the lack of a retinal biopsy in this case limits our understanding of the underlying pathophysiologic mechanisms, certain drugs and trauma are possible causes of secondary retinal pigmentary lesions. However, secondary retinal pigmentary lesions can be caused by certain medications or trauma, none of which were present in this case, so we concluded that the symptoms were related to ALL. To the best of our knowledge, there are no reports of secondary retinitis pigmentosa due to leukaemia. In the present case, this ocular finding was observed during the remission phase of leukaemia; however, subsequent detection of leukaemia cells in the bone marrow resulted in a molecular genetic relapse state. In light of the aforementioned considerations, it is imperative to meticulously monitor the patient's subsequent course of treatment, as retinitis pigmentosa-like symptoms and other serious symptoms may manifest even during the remission phase. Moreover, we posit that the correlation between ocular manifestations and molecular genetic relapse necessitates further investigation with a larger sample size in the future.

With regard to the second lesson learned, the patient achieved molecular genetic remission at three years and nine months of age. However, two months later, findings consistent with retinitis pigmentosa were observed. However, regular follow-up was conducted, which permitted prompt intervention. As evidenced by a previous study, prompt intervention for ophthalmic manifestations associated with leukaemia is crucial for maintaining optimal visual function [3]. A previous study has also indicated that ophthalmic manifestations are a significant factor in evaluating the progression of leukaemia [13]. Moreover, an additional study indicates that, in addition to ophthalmic manifestations, other symptoms, such as fever, anaemia, petechiae, and bone and joint pain, may occur during the remission phase of leukaemia [14]. Although the incidence of ocular lesions in children is lower than in adults, diagnosis may be delayed because children are less likely to report subjective symptoms [15,16]. In light of the above discussion, this case emphasizes the importance of conducting regular follow-up in patients with leukaemia from the perspective of various medical departments, including ophthalmologists.

## Conclusions

This case report documents the observation of retinitis pigmentosa-like fundus findings in a paediatric patient with ALL in remission. In conclusion, we emphasise that ophthalmic manifestations can occur even during the remission phase of leukaemia. In addition, we emphasise the potential importance of regular multidisciplinary follow-up to address the various symptoms associated with leukaemia, including ophthalmic manifestations. To put the above into practice, we believe that a multidisciplinary team approach involving ophthalmologists is necessary for the management of leukaemia patients. Finally, this case highlights the importance of ophthalmologic monitoring in pediatric leukaemia patients and its clinical significance.
